# Higher-achieving children are better at estimating the number of books at home: Evidence and implications

**DOI:** 10.3389/fpsyg.2022.1026387

**Published:** 2022-10-18

**Authors:** Kimmo Eriksson, Jannika Lindvall, Ola Helenius, Andreas Ryve

**Affiliations:** ^1^School of Education, Culture and Communication, Mälardalen University, Västerås, Sweden; ^2^Institute for Futures Studies, Stockholm, Sweden; ^3^Department of Pedagogical, Curricular and Professional Studies, University of Gothenburg, Göteborg, Sweden

**Keywords:** estimation skills, socioeconomic status, achievement gaps, differential reliability, human development

## Abstract

The number of books at home is commonly used as a proxy for socioeconomic status in educational studies. While both parents’ and students’ reports of the number of books at home are relatively strong predictors of student achievement, they often disagree with each other. When interpreting findings of analyses that measure socioeconomic status using books at home, it is important to understand how findings may be biased by the imperfect reliability of the data. For example, it was recently suggested that especially low-achieving students tend to underestimate the number of books at home, so that use of such data would lead researchers to overestimate the association between books at home and achievement. Here we take a closer look at how students’ and parents’ reports of the number of books at home relate to literacy among fourth grade students, by analyzing data from more than 250,000 students in 47 countries participating in 2011 PIRLS. Contrary to prior claims, we find more downward bias in estimates of books at home among high-achieving students than among low-achieving students, but unsystematic errors appear to be larger among low-achieving students. This holds within almost every country. It also holds between countries, that is, errors in estimates of books at home are larger in low-achieving countries. This has implications for studies of the association between books at home and achievement: the strength of the association will generally be underestimated, and this problem is exacerbated in low-achieving countries and among low-achieving students.

## Introduction

Students with a socioeconomically more advantaged family background tend to achieve better in school (White, 1980; Sirin, 2005; Harwell et al., 2017). This socioeconomic achievement gap is observed across various operationalizations of SES, such as parental education and occupation and wealth possessions (Mullis et al., 2009; Marks and O’Connell, 2021). In the present study we focus on the number of books at home, which has been considered an attractive measure of SES for several reasons, including relatively high correlations with parents’ income and education (Beaton et al., 1996), high response rates (Wiberg and Rolfsman, 2021), and that no laborious coding of the data is required (Heppt et al., 2022). The number of books at home variable is therefore commonly used in studies of the socioeconomic achievement gap (Eriksson et al., 2020) or to control for SES when gauging the relationship between achievement and other important educational factors such as learning opportunities (Yang Hansen and Strietholt, 2018; Rolfe et al., 2021). The number of books at home is also frequently used to complement other socioeconomic measures. For example, the socioeconomic index in the OECD Programme for International Student Assessment (PISA) is based on several indicators, including the number of books at home (Avvisati, 2020).

Many authors have noted that the number of books at home is a particularly strong predictor of student achievement (e.g., Hanushek and Woessmann, 2011; Brese and Mirazchiyski, 2013). One interpretation is that the number of books at home is an indicator of a family interest in reading, which is considered a factor that promotes achievement (Ammermueller and Pischke, 2009).

Because of the wide usage of the books at home variable, it is important that researchers examine the reliability of the variable. Prior studies, using datasets that include both students’ and their parents’ reports of the number of books at home have indicated that the reliability is not very good, because the correlation between student-reported and parent-reported data is often quite low and never very high (Rutkowski and Rutkowski, 2010, 2018; Jerrim and Micklewright, 2014). While reporting errors are likely found among both students and parents, it is commonly assumed that reliability is a greater concern for student reports (Engzell, 2021). The topic of the present paper is how data reliability may systematically vary with students’ achievement level.

### A hypothesis about variation in reliability

From a cognitive perspective, estimation of the number of books at home is a non-trivial numerical task. We expect the accuracy of estimates of books at home to depend on how skilled the person is at numerical estimation tasks in general. Numerical estimation skills are counted among mathematical skills that are tested in international large-scale assessment (Schleicher et al., 2009). Hence, we expect estimates of books at home to be more reliable among high-achievers than low-achievers in mathematics. Moreover, high-achievers in math also tend to be high-achievers in reading literacy (Ding and Homer, 2020). Thus, literacy scores should be useful as a proxy for estimation skills. Our hypothesis is therefore that estimates of the number of books at home are less reliable among low-achievers than high-achievers in school, regardless of whether achievement is measured in math or reading.

This hypothesis seems not to have been stated before. However, a prior study found higher literacy scores among students who reported the same number of books at home as their parents did than among students whose estimates deviated from their parents’ (Jerrim and Micklewright, 2014). While this result is what we would expect from our hypothesis on how reliability varies with achievement, it is not the correct comparison to make to properly test our hypothesis. For example, it could be that high-achievers seldom make errors but that any errors they do make tend to be large, in which case their reliability could still be poorer than among low-achievers. Testing the hypothesis requires an explicit comparison of reliability between low-achievers and high-achievers.

### A hypothesis about variation in bias

Errors may be random or systematic, also known as bias. A recent study claimed that estimates are biased downward especially among low-achieving students (Engzell, 2021), based on the finding that low-achieving students report having fewer books than high-achieving students do when the number of books at home reported by parents is held fixed. However, this finding can be explained without any downward bias among low-achieving students. It is sufficient that low-achieving students truly tend to have fewer books than high-achieving students, because this basic association will be observed also when parents’ estimates of books at home are held constant as the true numbers will still vary (due to the presence of unsystematic errors among parents).

There is in fact reason to expect the opposite to Engzell’s claim, that is, we expect that estimates are biased downward especially among *high-achieving* students. The reason is that estimates of books at home are made on a scale with a lowest step (0–10 books) and a highest step (more than 200 books). If the true number of books at home is at the lowest step, the only possible error in data is to make an overestimation. Because of the basic association between the true number of books at home and achievement, the true number of books at home is more often at the lowest step for low-achievers than for high-achievers. Thus, the existence of a lowest step of the response scale should cause more overestimation among low-achievers than high-achievers. Similarly, when the true number of books is at the highest step, which will happen more often for high-achievers than low-achievers, the only possible error in data is to make an underestimation. For these reasons, we expect more downward bias among high-achievers than low-achievers.

### Estimating the association between books at home and achievement

Many scholars are interested in how the association between socioeconomic status and student achievement varies across countries (e.g., Van de Werfhorst and Mijs, 2010; OECD, 2018; Kim et al., 2019; Strietholt et al., 2019). Surprisingly, results may strongly depend on how socioeconomic status is operationalized. For example, a recent study found that the association between wealth and achievement is stronger in *less* developed societies, whereas the association between books at home and achievement is stronger in *more* developed societies (Eriksson et al., 2021). Here we propose that this paradoxical finding may partly be due to how the reliability of books at home data varies across countries. To see why, consider the following points:

1. In a global comparison, low-developed countries tend to have more low-achieving students (e.g., Mullis et al., 2012; Stoet and Geary, 2013; Eriksson et al., 2020).
2. Earlier we hypothesized that students’ achievement level serves as a proxy for their numerical estimation skills and hence that more low-achieving students will produce less reliable estimates of books at home. In countries with lower achievement levels, we would therefore expect generally lower reliability in data on books at home.
3. Low reliability will attenuate the association between books at home and achievement, that is, make it look weaker than it really is.

**Figure 1:**
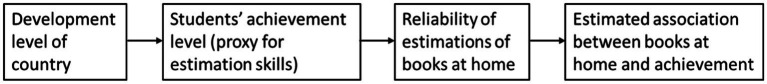
An explanation for why the estimated association between books at home and achievement is stronger in more developed countries.

Consequently, we expect underestimation of the association between books at home and achievement to be exacerbated in low-achieving countries. The association between books at home and student achievement is therefore expected to be weaker in low-developed countries simply due to less reliable data. This pathway is illustrated in Figure 1.

### An assumed relation between students’ and parents’ reliability

Absent data on the true number of books at home, we cannot say what the error is in individual estimates. Instead, we will assess the reliability at group level. Specifically, we use the strength of the correlation between students’ estimates and their parents’ estimates in a given group (e.g., the group of low-achieving students in a certain country). Our working assumption is that the estimation skills of students and parents are correlated, due to their shared genes and shared environment. Thus, when comparing reliabilities across groups, we take the correlation between students’ and parents’ estimates of books at home in a group as a proxy not only for the reliability of student data but also for the reliability of parent data. In other words, a relatively low correlation in a group is assumed to mean that both students and parents in this group make relatively unreliable estimates.

### Outline of study

As explained above, we use the correlation between students’ and parents’ estimates in each group as a measure of the reliability of their estimates of books at home. Using these measures, we test our hypotheses (1) that data reliability is lower among low-achieving students than among high-achieving students in the same country, and (2) that data reliability is lower in low-achieving countries than in high-achieving countries. We further use mediation analyses to examine whether the latter hypothesis can also explain why the association between achievement and books at home is weaker in less developed countries.

We then address the question of whether there is a difference in bias between the estimates of high- and low-achieving students within countries by examining whether the two groups of students differ in how their estimates deviate from their parents’. Here we assume that the bias of *parents* of high- and low-achieving students differs less than the bias of the students themselves, which seems very reasonable as errors are generally assumed to be generally smaller among parents than students (Engzell, 2021).

Finally, given its reliability issues, one may question whether it is worthwhile to study the books-at-home variable at all. To demonstrate that this variable taps into something important, we show that parent-reported data on books at home predict literacy above and beyond parents’ education and occupation.

## Materials and methods

Following Engzell (2021), we test our hypotheses using publicly available data from the 2011 wave of PIRLS.^1^ The details of this assessment are described elsewhere (Mullis et al., 2009).

### Countries

We include data from 47 participating countries and country-like entities in the 2011 wave of PIRLS: Australia, Austria, Azerbaijan, Belgium, Botswana, Bulgaria, Canada, Colombia, Croatia, Czech Republic, Denmark, Finland, France, Georgia, Germany, Honduras, Hong Kong, Hungary, Indonesia, Iran, Ireland, Israel, Italy, Kuwait, Lithuania, Malta, Morocco, Netherlands, New Zealand, Northern Ireland, Norway, Oman, Poland, Portugal, Qatar, Romania, Russia, Saudi Arabia, Singapore, Slovak Republic, Slovenia, South Africa, Spain, Sweden, Taiwan, Trinidad and Tobago, United Arab Emirates.

### Samples

The PIRLS target population is the grade that represents 4 years of schooling. The average age of students is typically between 10 and 11 years. Representative samples of students are drawn in each country. All participating students from the 47 countries are included in our study. The number of participating students per country ranged from 3,349 in Hong Kong to 18,245 in Canada. The total number of participants is 307,747. In line with the representativity of the samples, the gender distribution is almost perfectly even: 50.5% boys and 49.5% girls. The sampling scheme and country samples are described in more detail elsewhere (Mullis et al., 2009, 2012).

### Measures

To measure reading literacy, PIRLS ask students to read certain texts and answer questions about them. A rotated booklet design is used whereby every student reads only a few of the full set of texts. PIRLS then imputes a set of plausible values for the student’s score on the full test. IEA provides software for analysis that accounts for the additional uncertainty added by this design,^2^ as well as sampling weights, etc. We use this software to calculate all the measures used in the further analysis: average achievement scores per country, reliability measures, and the association between books at home and achievement.

The questionnaire to students participating in PIRLS includes the question “About how many books are there in your home? (Do not count magazines, newspapers, or your school books.)” There are five response options: None or very few (0–10 books); Enough to fill one shelf (11–25 books); Enough to fill one bookcase (26–100 books); Enough to fill two bookcases (101–200 books); Enough to fill three or more bookcases (more than 200). These options are coded from 1 to 5. A similar question, but excluding children’s books, is included in the questionnaire to parents of participating students: “About how many books are there in your home? (Do not count ebooks, magazines, newspapers, or children’s books.)” The five response options are the same intervals as in the question to students, that is, 0–10, 11–25, 26–100, 101–200, and more than 200. These options are coded from 1 to 5. From the questionnaire to parents, we also obtain data on parents’ highest levels of education and occupation.

Finally, the development level of a country is operationalized by the Human Development Index (HDI), available from the United Nations Development Programme^3^ for 45 countries in our study. We use HDI values obtained from other sources for Taiwan^4^ and Northern Ireland.^5^

### Data analysis

The first analysis concerns within-country differences in reliability. We perform a median split of the student sample in each country, based on their literacy score (operationalized as the average of the plausible values available for the student). In each half of the sample, we use the Pearson correlation between students’ and parents’ data on books at home as a measure of the reliability of the data in that group. This yields two reliability measures per country: one measure for below-median achievers and one measure for above-median achievers. We then compare these measures using a paired *t*-test.

The second analysis section concerns between-country differences in reliability and other variables involved in the pathway depicted in Figure 1. We use the Human Development Index as a measure of the development level of each country. We use the mean literacy score in each country as a measure of the achievement level in that country. We use the Pearson correlation between students’ and parents’ data on books at home, calculated separately in each country, as a measure of the reliability of the data in that country. We further use the Pearson correlation between students’ literacy scores and their estimates of the number of books at home, calculated separately in each country, as a measure of the strength of the association between literacy and books at home data from students, and similarly for parents’ data. We calculate pairwise correlations between these country-level measures to examine the links of the pathway in Figure 1. We then perform a formal mediation analysis.

A third analysis concerns how the bias in students’ estimates of the number of books at home varies their literacy within countries. In analogy with the first analysis above, we perform a median split of the student sample in each country based on literacy scores. In each group, we use the mean difference between students’ and parents’ estimates as a measure of the bias in students’ data. This yields two bias measures per country, one measure for below-median achievers and one measure for above-median achievers, which we compare using a paired *t*-test.

In a final analysis section, we examine how parent-reported books at home fare as a predictor of literacy compared to the other parent-reported socioeconomic variables in PIRLS: parents’ highest education and parents’ highest occupation. We first compare how different socioeconomic variables correlate with literacy scores. We then use multiple linear regression to examine whether the number of books at home predicts student literacy above and beyond parents’ education and occupation.

## Results

### The reliability of students’ estimates varies with their achievement level

Across 47 countries, reliability measures were lower for the groups of below-median achievers, *M* = 0.38 (*SD* = 0.12), than for the groups of above-median achievers, *M* = 0.50 (*SD* = 0.08), a difference of 0.12, 95% CI [0.09, 0.14], *t*(46) = 9.02, *p* < 0.001, paired *t*-test. Thus, the hypothesis that lower-achieving students make larger unsystematic estimation errors was supported.

**Figure 2:**
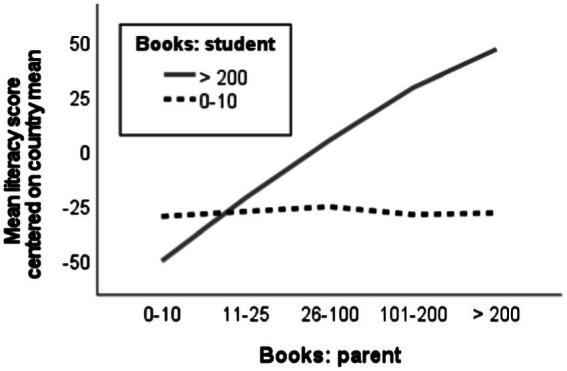
How mean literacy scores (centered on the mean in each country) among students who reported the lowest number of books at home (0–10, dashed line) or the highest number of books at home (> 200, solid line) vary across different values of books at home as reported by parents.

A consequence of this hypothesis is that a large difference between the estimates of student and parent indicates that the student is probably a low achiever. To illustrate this phenomenon, Figure 2 shows what the association between literacy scores and parent-reported books at home looks like among students who themselves report either the lowest (0–10) or the highest (> 200) number of books at home. In these groups of students, their estimation error will tend to have very different relations to the number of books reported by parents. Namely, the more books at home that parents report, the more inaccurate we expect the lowest student estimates to be, and the less inaccurate we expect the highest student estimates to be. Among students who report the lowest number of books, the graph in Figure 2 is flat, consistent with a negative association between literacy and estimation inaccuracy that offsets the positive association between literacy and books at home. Among students who report the highest number of books, by contrast, the graph starts very low and increases very steeply, consistent with estimation inaccuracy now changing in the opposite direction so that the two associations reinforce each other.

### Tests of the country-level hypotheses

Correlations between country-level variables are reported in Table 1. These correlations support all the links of the pathway depicted in Figure 1. First, the development level of countries is strongly correlated with their achievement level. Second, the achievement level is strongly correlated with the reliability of books at home data. Third, the reliability of books at home data is strongly correlated with the strength of the association between literacy and books at home, whether estimated by students or parents.

**Table 1:** Country-level correlations.

	1	2	3	4	5
1. Human Development Index	–				
2. Mean literacy score	0.74 [0.56,0.84]	–			
3. Reliability of books at home data	0.32 [0.03,0.55]	0.60 [0.37,0.76]	–		
4. Association btw. Literacy and books at home data from students	0.42 [0.15,0.63]	0.65 [0.44,0.79]	0.79 [0.65,0.88]	–	
5. Same for data from parents	0.28 [−0.01,0.52]	0.42 [0.14,0.62]	0.76 [0.60,0.86]	0.80 [0.66,0.88]	–

Based on *n* = 47 countries. 95% confidence intervals based on Fisher’s r-to-z transformation with bias adjustment.

For sequential mediation analysis we use Model 6 of the PROCESS macro for SPSS (Hayes, 2017) to calculate 95% confidence intervals for indirect effects using 5,000 bootstrap samples. The results, in Table 2, indicate that the path *via* a country’s mean literacy level and reliability level indeed produces a considerable indirect effect of the development level on the association between literacy and books at home, whether estimated using data from students or parents. The effects of other paths were not statistically significant, that is, their confidence intervals include zero. Thus, the hypothesis illustrated in Figure 1 was supported.

**Table 2:** Results of sequential mediation analysis of the effect of HDI on the strength of the association between students’ literacy scores and estimates of their number of books at home.

Path	Effect, student data	Effect, parent data
HDI → Mean literacy → Reliability → Association	0.50 [0.24, 0.93]	0.50 [0.26, 0.88]
HDI → Mean literacy → Association	0.21 [−0.16, 0.57]	−0.16 [−0.50, 0.12]
HDI → Reliability → Association	−0.23 [−0.64, 0.07]	−0.23 [−0.65, 0.06]
Direct effect	0.08 [−0.27, 0.42]	0.18 [−0.13, 0.48]
Total indirect effect	0.47 [0.08, 0.91]	0.11 [−0.29, 0.46]

Based on *n* = 47 countries. 95% confidence intervals based on 5,000 bootstrap samples. To obtain more convenient numbers, literacy scores were scaled down by a factor of 1,000 in this analysis.

The mediation analysis reported in Table 2 is based on a series of linear regressions. We report these underlying analyses in the case of student data. First, mean literacy is regressed on HDI, yielding a positive effect estimate *B* = 0.53, 95% CI [0.38, 0.67], *p* < 0.001. This is the first arrow in Figure 1. Second, reliability is regressed on both mean literacy and HDI, yielding a positive effect of mean literacy, *B* = 1.42 [0.80, 2.03], *p* < 0.001, but no significant direct effect of HDI, *B* = −0.35, [−0.79, 0.09], *p* = 0.12. This means that the effect of HDI on reliability follows the path formed by the first two arrows in Figure 1. Third, the association between literacy and student-reported books at home is regressed on reliability, mean literacy, and HDI, yielding a positive effect of reliability, *B* = 0.67 [0.43, 0.90], *p* < 0.001, but no significant direct effect of mean literacy, *B* = 0.40 [−0.17, 0.99], *p* = 0.17, or of HDI, *B* = 0.08, [−0.27, 0.42], *p* = 0.65. This means that the effect of HDI on the strength of the association follows the path formed by the three arrows in Figure 1.

### Downward bias in books at home data is stronger among high-achievers

Across the 47 countries, there was stronger downward bias in students’ estimates of books at home in the groups of above-median achievers *M* = −0.16 (*SD* = 0.21), than in the groups of below-median achievers, *M* = −0.06 (*SD* = 0.23), a difference of −0.10, 95% CI [−0.13, −0.06], *t*(46) = 5.66, *p* < 0.001, paired *t*-test. Thus, the hypothesis that downward bias in books at home data is stronger among high-achieving students than low-achieving students was supported.

### The number of books at home predicts students’ literacy above and beyond other socioeconomic measures

From the results in Table 2, we conclude that the association between books at home and literacy is attenuated, due to poor reliability, even when books at home data are reported by parents. Despite the attenuation, the strength of the association between literacy and parent-reported books at home is comparable with the strength of the associations between literacy and parents’ highest education and occupation (reported by the parents themselves). In the average country, the correlations with literacy are between 0.30 and 0.36 for the three socioeconomic variables, see Table 3.

**Table 3:** Mean values of within-country correlations.

	Literacy	Books at home	Parents’ education
Books at home	0.31 [0.28, 0.33]		
Parents’ education	0.36 [0.34, 0.39]	0.42 [0.39, 0.45]	
Parents’ occupation	0.30 [0.28, 0.33]	0.36 [0.33, 0.39]	0.56 [0.53, 0.58]

Based on *n* = 47 countries. 95% confidence intervals within brackets.

**Figure 3:**
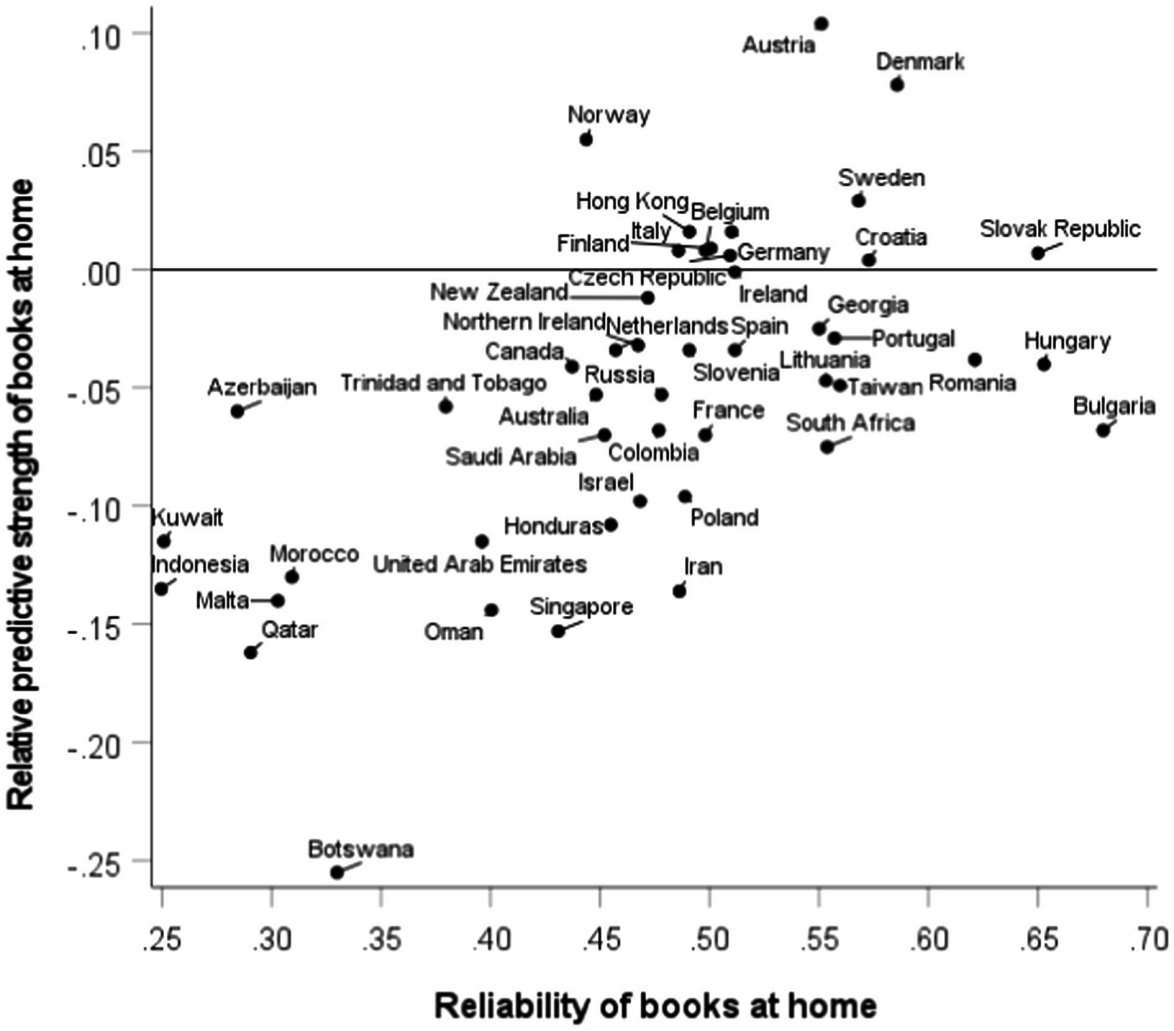
Country variation in the reliability of books at home data (x-axis) and the relative predictive strength of books at home (y-axis), operationalized as the difference between the literacy-books at home correlation and the literacy-parents’ education correlation. Above the reference line at zero are 12 countries where student literacy was better predicted by books at home than by parent’s education.

Which of the three socioeconomic variables had the strongest correlation with literacy varied across countries. In some countries it was books at home (12 countries), but most often it was parents’ education (34 countries). However, recall that the correlation with books at home is attenuated by low reliability, which also varies across countries. This is illustrated by a scatter plot in Figure 3. The x-axis shows our measure of the reliability of books at home data in each country. The y-axis shows the relative predictive strength of books at home, measured by the difference in strength between the literacy-books at home correlation and the literacy-education correlation. Note that there are 12 countries above the reference line at zero. The plot shows a strong positive correlation between the reliability of books at home and its relative predictive strength, *r* = 0.59, 95% CI [0.36, 75], *p* < 0.001. This finding suggests that if books at home could be measured more reliably, it is likely that it would more generally be the strongest socioeconomic predictor of literacy.

To drive home the point that the number of books at home predicts literacy above and beyond other socioeconomic variables, we also report multiple regression analyses with parents’ books at home data, parents’ highest level of education, and parents’ highest occupation as simultaneous predictors of student literacy. As shown in Table 3, these variables are intercorrelated, but not so strongly that multicollinearity is a problem. Multiple regression analyses, performed separately in each country, yielded three standardized coefficients per country: β_books_, β_education_, and β_occupation_. These coefficients were generally positive and statistically significant at the *p* < 0.05 level; exceptions were one country in which β_books_ was not significantly positive, and eight countries in which β_occupation_ was not significantly positive. The average country had β_books_ = 0.16, 95% CI [0.15, 0.17], β_education_ = 0.21, 95% CI [0.19, 0.22], and β_occupation_ = 0.11, 95% CI [0.09, 0.12]. We conclude that the number of books at home in general has a considerable effect on literacy above and beyond parents’ education and occupation, even when attenuated by low reliability.

## Discussion

### Why study books at home?

The number of books at home is a commonly used proxy of students’ socioeconomic status in educational studies. One reason is that this variable is present in all international large-scale studies, which makes it easy to compare results across data sources (Blömeke et al., 2016). On the other hand, several studies have pointed out issues with the reliability of books at home data (Rutkowski and Rutkowski, 2010, 2018; Jerrim and Micklewright, 2014; Engzell, 2021). Should the variable therefore be abandoned? We do not think so, because the number of books appears to tap into an especially important aspect of students’ family background that goes beyond other common socioeconomic variables such as parents’ education and occupation (Eriksson et al., 2021). In support of this notion, many authors have noted that the number of books at home is a particularly strong predictor of student achievement (e.g., Hanushek and Woessmann, 2011; Brese and Mirazchiyski, 2013). However, Engzell (2021) pointed out a problem with this interpretation and argued that the strength of the association between student-reported books at home and achievement is an artifact of reverse causality in the form of a tendency among high-achieving students to acquire more books (Engzell, 2021).

To shed more light on this question, we studied the association with parent-reported books at home. Parents are asked to exclude children’s books in their estimates so their data should not suffer from the reverse causality problem. In our analysis, we nonetheless found that the number of books at home that parents report predicts their children’s literacy score above and beyond parents’ education and occupation. Our conclusion is that the true number of books at home has an important and unique association with the literacy of fourth grade students. One interpretation is that parents’ interest in reading is transferred to students, either socially or *via* genetic transfer, and that interest in reading is beneficial for academic achievement (Ammermueller and Pischke, 2009; Eriksson et al., 2021). We believe that more research needs to be devoted to testing this explanation, and other possible explanations, for the association between books at home and achievement. For this reason, we believe researchers should not refrain from making use of available estimates of books at home, despite their reliability issues. Our recommendation is instead that researchers be careful about taking reliability issues into account when interpreting results.

### Taking the relation between reliability and achievement into account

The main aim of the current study was to draw attention to the issue that the reliability of books at home data varies systematically across achievement levels. We find that data reliability is lower among lower-achieving students as well as in lower-achieving countries. A plausible explanation is that students who achieve better in school tend to have better numerical estimation skills. This issue has implications for studies that use the number of books at home to control for family background when studying the effect of another variable on student achievement (e.g., Blömeke et al., 2016; Eriksson et al., 2019; Karadavut et al., 2019; Wennström, 2020). Poor reliability implies that the true number of books is not fully controlled for in such studies, and the problem of insufficient control will be especially bad in low-achievement countries and among low-achieving students.

There are also implications for studies that use the number of books at home to measure the size of the socioeconomic achievement gap. In a recent meta-analysis, Harwell et al. (2017) called these gaps “surprisingly modest.” However, low reliability of data typically means that the size of achievement gaps will be underestimated. This underestimation of achievement gaps will be most pronounced in low-achievement countries. Lack of awareness of this phenomenon may lead researchers to unnecessarily look for other explanations. For example, several prior studies have observed a stronger association between books at home and academic achievement in more developed countries, and they have proposed explanations in terms of the use of books or the access to books (Chiu, 2010; Eriksson et al., 2021). Our study indicates that the real explanation why the association is stronger in more developed countries is that in these countries we should expect estimation skills to be higher. Hence, books-at-home data will be more reliable and yield stronger associations with achievement in more developed countries.

Our finding also means that studies of achievement gaps in different groups within a country will tend to underestimate gaps especially in lower-achieving groups. For example, consider prior findings of a weaker association between student achievement and books at home among students with immigrant background than among non-immigrant students in England and Sweden (Elmeroth, 2006; Hansson and Gustafsson, 2013; Lenkeit et al., 2015). Such findings may be artifacts of differences in the reliability of books at home data, as it is likely that immigrants also tend to have overall lower achievement levels and hence provide data of lower reliability.

### Does bias in estimates of books at home vary with the achievement level?

Another possible issue with estimates of books at home is that they may be biased in some direction. Engzell (2021) claimed that estimates are biased downward among low-achieving students, but this finding appears to have been an artifact of the analysis method that was used. In our analysis, comparing students’ and parents’ estimates, we found more downward bias among high-achievers than low-achievers.

### Limitations

A limitation of our study (and of all studies in this area) is that, lacking data on the true number of books at home, we cannot tease apart errors in students’ estimates from errors in parents’ estimates. To get around this problem, we focused on group level comparisons. We assumed that the estimation skills of parents and students are correlated, especially at group level (e.g., countries with weaker school systems are expected to have lower estimation skills both in the parents’ generation and the children’s generation). To measure the overall reliability of estimates in a group, we used the correlation between students’ and parents’ estimates. If our assumption is correct, this measure of overall reliability will, across groups, simultaneously capture variation in the reliability of students’ and parents’ estimates. Consistent with our assumption, we found that the reliability measure in a country is a very strong predictor of the strength of the association between literacy and books at home, whether estimated by parents or students.

In this paper we do not present any equations; our hypotheses were motivated by verbal arguments. The same hypotheses could alternatively be derived in a more formal way, that is, we could formulate a formal model of estimation errors that depend on achievement, fit this model to existing data, and show that simulated data from the fitted model support the same hypotheses.

## Conclusion

The number of books at home is a valuable variable for researchers seeking to understand how family background influences children’s literacy—but this variable has specific reliability issues that researchers need to be aware of to avoid incorrect interpretations of data. It is not possible to quantify how researchers should adjust findings obtained using data on the number of books at home. Qualitatively, though, researchers should expect that observed associations between books at home and achievement (or any other variable) are weaker than the true associations, especially in lower-achieving group of students.

## Data availability statement

Publicly available datasets were analyzed in this study. This data can be found here: Data from the 2011 wave of PIRLS are available at: https://timssandpirls.bc.edu. The Human Development Index is available from the United Nations Development Programme (http://hdr.undp.org/) for 45 countries in our study; we use HDI values obtained from other sources for Taiwan (https://www.dgbas.gov.tw) and Northern Ireland (https://globaldatalab.org).

## Ethics statement

Ethical review and approval was not required for the study on human participants in accordance with the local legislation and institutional requirements. Written informed consent from the participants’ legal guardian/next of kin was not required to participate in this study in accordance with the national legislation and the institutional requirements.

## Author contributions

KE conceived of the study, performed the analysis, and wrote the paper. JL, OH, and AR contributed to the survey of the literature and to the discussion, and provided critical feedback on the manuscript. All authors contributed to the article and approved the submitted version.

## Funding

This work was supported by the Swedish Research Council under Grant 2014–2008.

## Conflict of interest

The authors declare that the research was conducted in the absence of any commercial or financial relationships that could be construed as a potential conflict of interest.

## Publisher’s note

All claims expressed in this article are solely those of the authors and do not necessarily represent those of their affiliated organizations, or those of the publisher, the editors and the reviewers. Any product that may be evaluated in this article, or claim that may be made by its manufacturer, is not guaranteed or endorsed by the publisher.

## Footnotes

^1^ https://timssandpirls.bc.edu

^2^ https://www.iea.nl/data-tools/tools

^3^ http://hdr.undp.org/

^4^ https://www.dgbas.gov.tw

^5^ https://globaldatalab.org
